# Pioglitazone Enhances Cytosolic Lipolysis, β-oxidation and Autophagy to Ameliorate Hepatic Steatosis

**DOI:** 10.1038/s41598-017-09702-3

**Published:** 2017-08-22

**Authors:** Pi-Jung Hsiao, Hsin-Ying Clair Chiou, He-Jiun Jiang, Mei-Yueh Lee, Tusty-Jiuan Hsieh, Kung-Kai Kuo

**Affiliations:** 1 Division of Endocrinology and Metabolism, Department of Internal Medicine, Kaohsiung Medical University Hospital, Kaohsiung City, Taiwan; 2 Graduate Institute of Medicine, Kaohsiung Medical University, Kaohsiung City, Taiwan; 3 Division of General and Digestive Surgery, Department of Surgery, Kaohsiung Medical University Hospital, Kaohsiung City, Taiwan; 4 School of Medicine, College of Medicine, Kaohsiung Medical University, Kaohsiung City, Taiwan

## Abstract

Non-alcoholic fatty liver disease closely contributes to the development of obesity and insulin resistance. Even though pioglitazone has been reported to effectively lessen hepatic steatosis in human studies, its molecular mechanism remains unclear. This study is designed to investigate the regulation of cytosolic lipolysis, β-oxidation and autophagy by pioglitazone in a mice model of high fat diet (HFD) and cell model incubated with palmitic acid. Our results revealed hepatic steatosis was apparently induced by HFD and it was significantly reversed by pioglitazone. The serum insulin and hepatic triglyceride content was significantly decreased by co-administered pioglitazone with HFD. Hepatic expression of cytosolic-lipolysis related proteins (ATGL, HSL), β-oxidation (CPT-1A) and autophagy-related proteins (ATG7, LC3, LAL) was significantly enhanced by pioglitazone. Knockdown PPARα/PPARγ in AML12 cells significantly and proportionally reduced the expressions of ATGL, CPT-1A and LC3II, which was induced by pioglitazone. Furthermore, facilitation of the autophagic flux by pioglitazone was obviously blocked by lysosomal inhibitor, leupeptin, to demonstrate accumulation of the LC3II and intracellular lipid in AML12 cells. Our results demonstrated that pioglitazone attenuating the hepatic steatosis may be mediated by enhancing cytosolic lipolysis, β-oxidation and autophagy in a PPARα and PPARγ dependent manner.

## Introduction

Fatty liver disease (FLD), defined as fat accumulation exceeding 5 to 10% of the liver weight, is probably the most common etiology (~25%) of chronic liver diseases in the West and Asia^1, 2^. In the past, alcoholic fatty disease accounted for the majority of FLD. But over-nutrition caused by more consumption of fat or simple sugar is currently an important cause to the burdensome epidemic of obesity and non-alcoholic fatty liver disease (NAFLD)^3, 4^. Hepatic fat is physiologically balanced by peripheral fat influx from the plasma non-esterified free fatty acid (NEFA) pool, dietary fat intake, de novo lipogenesis, β-oxidation by mitochondria, secreted as VLDL particles and intracellular lipolysis of triglycerides (TG)^5^. Imbalance of the fat metabolism leading to hepatic fat accumulation may be secondary to increased dietary fat from a HFD, increased lipogenesis by enhancing activity of lipogenic enzymes, acetyl CoA carboxylase (ACC) and fatty acid synthase (FAS)^6, 7^.

Lipolysis, a process of hydrolysis of triglyceride and cholesterol ester, is mainly determined by adipose triglyceride lipase (ATGL), hormone-sensitive lipase (HSL) and lysosomal acid lipase (LAL). TG contained in lipid droplets (LDs) is hydrolyzed by cytosolic lipases, ATGL and HSL, to generate free fatty acid for β -oxidation or packaged as very-low density lipoprotein particles for secretion^8^. Then, carnitine palmitoyl transferase (CPT-1A), the pivotal regulator of β-oxidation, drives fatty acid to go through inner mitochondrial membrane for metabolism^9^. LAL, highly expressed in hepatocytes and Kupffer cells, is essential for the hydrolysis of triglycerides and cholesteryl esters that are delivered to lysosomes via the LDL receptors or LDL-related proteins^10–12^. Autophagy is a degradation process of the intracellular components in lysosomes, determining cellular homeostasis maintenance. Macroautophagy is the most physiologically important mechanism of the lysosomal degradation system, which involves more than 30 proteins that generally known as autophagy-related proteins (Atg)^13, 14^. Autophagy-related proteins and their regulated processes are including (1) initiation by the inhibition of mammalian target of rapamycin (mTOR) and its active phosphorylated form (p-mTOR), (2) nucleation control by activation of the Beclin-1 related class III phosphatidylinositol 3-kinase (PI3K) complex (Vps 34), (3) vesicle elongation by Atg 7, (4) regulation of vesicle closure, phagolysosome fusion by microtubule-associated protein light chain 3 beta (soluble form LC3-I to lipidated form LC3-II), and finally lipolysis regulated by LAL^12, 15^. The hepatic degradation of LDs in lysosome, specified as “lipo (macro) autophagy” or lipophagy, is an alternative pathway for lipid degradation by LAL which mobilizes large amounts of lipid degradation rapidly^16, 17^.

Physiologically, ATGL determines the basal lipolysis and HSL entirely accounts for the stimulated lipolysis. Both ATGL and HSL are stimulated by catecholamine but inhibited by insulin in adipose tissue^8, 18^. Hepatic autophagy enhancement is naturally triggered by starvation or a short-term increase of lipid supply. Deregulation of autophagy has been proved to be a key process in developing hepatic steatosis^19–21^. The total proteome of liver is estimated to be degraded hourly from 1.5 to 5% under either fed or starved states. Autophagy is responsible for up to 70% of intracellular protein breakdown in liver^14^. Therefore, lipolysis plays a vital role to keep homeostasis of the intra-hepatic fat storage, especially the interplay of the lipolysome system between cytosolic lipolysis, β-oxidation and autophagy.

Pioglitazone is typically recognized as a peroxisome proliferator-activated receptor gamma (PPARγ) agonist clinically used as an insulin sensitizer. It has proved to possess hepatoprotection through mechanism of eliminating hepatic fat accumulation in human studies^22^. Although reduced free fatty acid influx and enhancing adiponectin have been proved to attenuate hepatic steatosis, it still could not clarify well how is the negative balance of hepatic steatosis by pioglitazone to alleviate hepatic fat storage. Since cytosolic lipolysis and autophagy are both negatively regulated by insulin to exhibit functional similarity^16, 18^, enhancing or activating either of the lipolysis is rationally supposed to treat hepatic steatosis of NAFLD and metabolic syndrome. In this study, we speculated that pioglitazone may enhance the cytosolic lipolysis and/or autophagy to attenuate the hepatic steatosis. To explore more, we designed a mouse model fed with HFD to mimic human NAFLD and test the molecular mechanism of by silent RNA assay in a cell model.

## Results

### HFD induced hepatic steatosis but ameliorated by co-administration of pioglitazone

**Figure 1:**
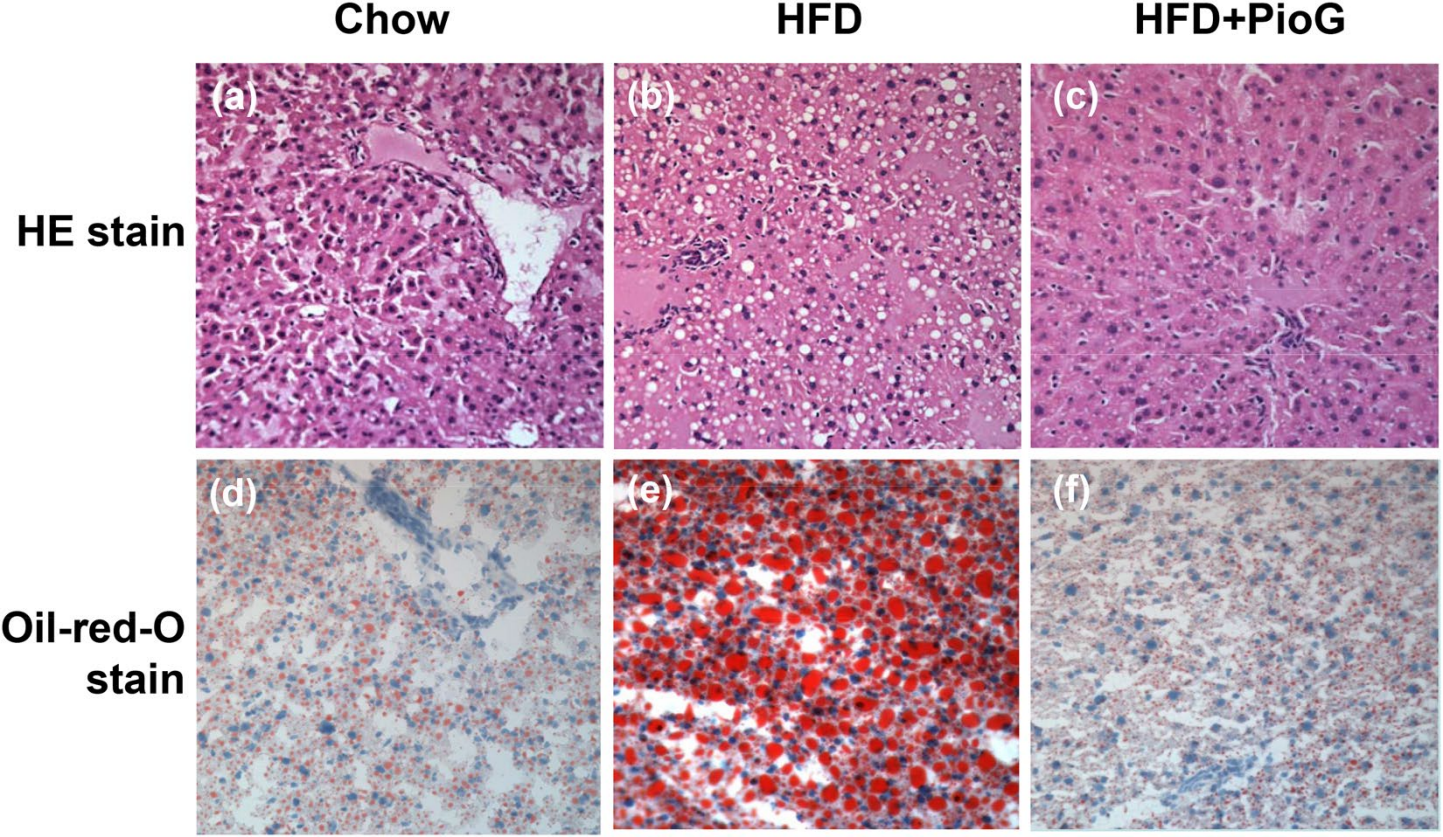
High fat diet-induced hepatic steatosis was attenuated by Pioglitazone. The liver specimens of mice, fed respectively with chow diet (**a**,**d**), high fat diet (HFD, **b**,**e**), or high fat diet co-administered with pioglitazone (PioG, 100 mg/kg/d) (**c**,**f**) for 8 weeks, were applied for immunohistochemical staining by hematoxylin & eosin (H&E) and Oil-red O stain. Magnification: 400X.

Mice fed with HFD significantly increased hepatic lipid accumulation (Table 1). However, the co-administration of pioglitazone with HFD markedly reduced hepatic lipid content, serum ALT and insulin versus the HFD only groups. The immunohistochemical study on HE and Oil-red O stains also demonstrated the same tendency, the hepatic steatosis was decreased in HFD treated with pioglitazone versus HFD only (Fig. 1). Consistent improvement of the hepatic steatosis in histochemical and biochemical study demonstrated that pioglitazone could improve insulin resistance and ameliorate the hepatic steatosis caused by HFD in mice.

**Table 1:** Biochemical data among three groups.

8 weeks	Chow diet (n = 5)	High fat diet (n = 5)	High fat diet + PioG (n = 8)	*P*
BW gain (gm)	5.22 ± 2.44	7.64 ± 4.13	5.60 ± 2.50	0.475
Blood glucose (mg/dl)	141.8 ± 10.51	149.2 ± 10.40	153.5 ± 11.72	0.432
Insulin (μg/L)	2.02 ± 1.62	2.67 ± 1.60	0.66 ± 0.10Δ	0.024
TG (mg/dl)	63.73 ± 18.75	64.48 ± 15.41	53.06 ± 8.94	0.403
ALT (IU/dl)	11.28 ± 4.74	18.12 ± 8.88	9.78 ± 1.65Δ	0.061
NEFA (μEq/L)	598.67 ± 121.50	686.67 ± 143.38	630.40 ± 145.66	0.283
Hepatic TG (mg/g protein)	222.1 ± 122.3	517.3 ± 109.00*	235.6 ± 150.90Δ	0.005
Liver weight (gm)	1.06 ± 0.90	1.07 ± 0.08	1.03 ± 0.15	0.454

Values were indicated as means ± S.D.; ALT: alanine aminotransferase; NEFA: non-esterified free fatty acid; Hepatic TG: hepatic triglyceride content; PioG: pioglitazone.

Kruskal-Wallis test among three groups and Student’s t test between two groups were used for statistical analysis; p < 0.05 indicated significant. *vs chow diet, Δvs high fat diet.

### Pioglitazone significantly enhanced expression of cytosolic lipolysis and autophagy related genes and decreased lipogenesis genes in hepatocyte

**Figure 2:**
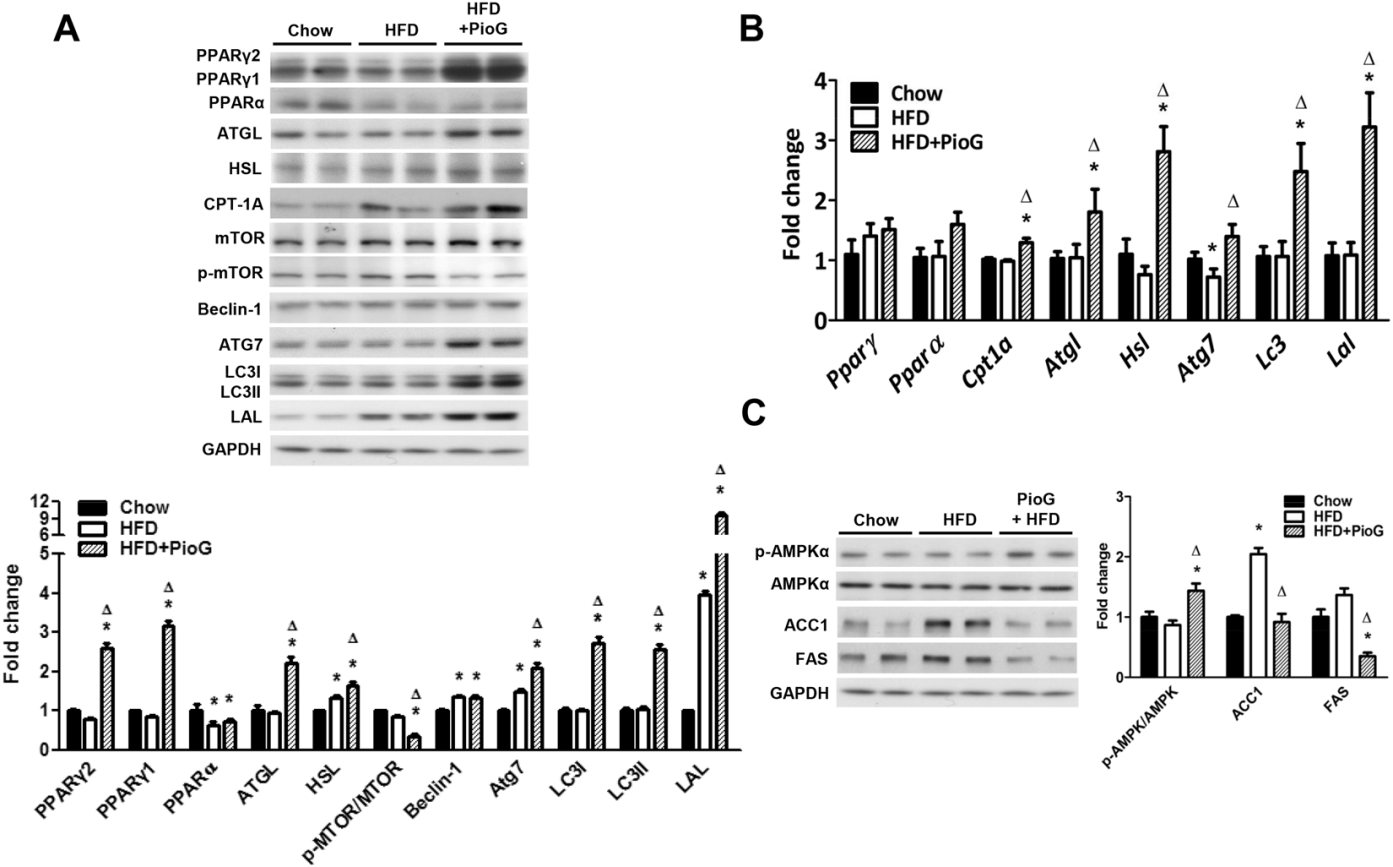
Pioglitazone enhanced cytosolic lipolysis, β-oxidation and autophagy but decreased lipogenesis. Mice were fed with chow diet, high fat diet, or high fat diet co-administered with pioglitazone (100 mg/kg/d) for 8 weeks. (**A**) Protein expressions related to cytosolic lipolysis (ATGL, HSL), β-oxidation (CPT-1A) and autophagy (mTOR, p-mTOR, Beclin-1, ATG7, LC3-I, LC3-II, and LAL) and the quantitative results. (**B**) Messenger RNA expressions of the above genes. (**C**) Protein expressions of the lipogenesis genes (FAS, ACC1) and the quantitative results. GAPDH served as a loading control. *P* < 0.05 indicated as significant, *vs chow diet, Δvs high fat diet.

Hepatic expressions of the PPARγ1 and 2 were significantly enhanced by pioglitazone (Fig. 2A). Proteins involved in cytosolic lipolysis (ATGL and HSL), and β-oxidation (CPT-1A) were also up-regulated by co-administration of pioglitazone with HFD. Autophagy related proteins from initiation, vesicle elongation, vesicle closure to final lysosomal lipolysis (p-mTOR, ATG7, LC3I, LC3II, and LAL) were also enhanced by adding pioglitazone with HFD compared with group of HFD. The lysosome specific LAL expression was four folds higher in group of HFD comparing with chow diet. But LAL expression was tremendously increased up to 10 folds by adding pioglitazone. In addition, messenger RNA expressions of the *Atgl, Hsl, Cpt-1A, Lc3, Atg7*, and *Lal* were significantly up-regulated by pioglitazone, as the result of transcriptional activation on these genes by pioglitazone (Fig. 2B). A HFD significantly increased expressions of lipogenesis related genes (FAS and ACC), but co-administered with pioglitazone significantly up-regulated phosphorylation of 5′-AMP-activated protein kinase (AMPKα) and down-regulated the gene expression for lipogenesis (Fig. 2C).

**Figure 3:**
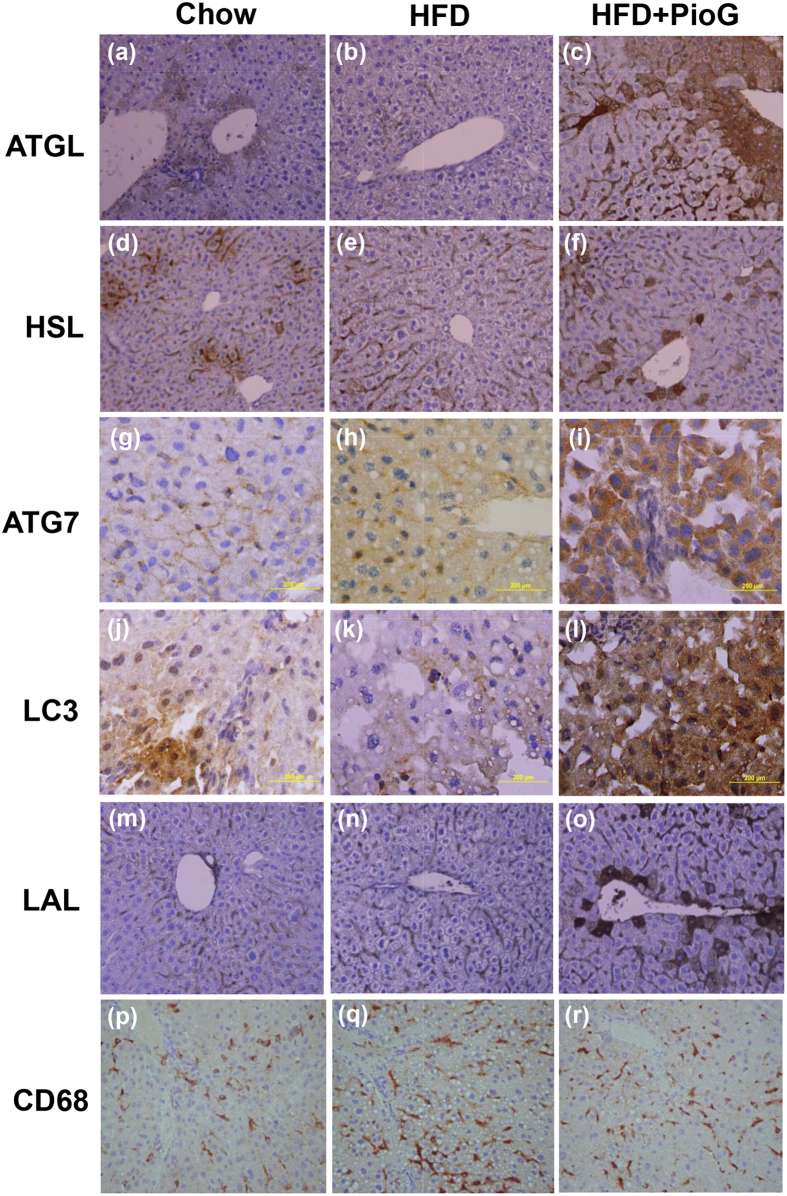
Pioglitazone increased immunochemical expressions of cytosolic lipolysis and autophagy-related proteins. The liver specimens were stained by antibodies against ATGL, HSL, ATG7, LC3, and LAL separately conditioned by chow diet, high fat diet, and high fat diet co-administered with pioglitazone (PioG, 100 mg/kg/d) for 8 weeks. Kupffer cell was characterized by CD68 staining. Magnifications, (**a**–**f**): 400X, (**g**–**l**): 1000X, (**m**–**o**): 40X, (**p**–**r**): 20X.

Expressions of the ATGL, HSL, ATG7, LC3, and LAL in mice liver were further evaluated among groups by immunohistochemical staining. Our results showed basal expression of the ATGL and HSL occurred in both hepatocytes and Kupffer cells on chow diet (Fig. 3a,d), where Kupffer cell in our liver specimens were characterized by mouse CD68 staining (Fig. 3p–r). HFD enhanced the expression of ATGL and HSL predominantly in Kupffer cells more than in hepatocytes (Fig. 3b,e). However, co-administered pioglitazone apparently augmented expressions of the ATGL and HSL to display mainly in hepatocytes (Fig. 3c,f). Expression of the ATG7 and LC3 were extremely increased by adding pioglitazone to HFD (Fig. 3g–i,j–l). Basal LAL expression occurred mainly in Kupffer cells both in group of chow and HFD diet, but expanded to hepatocytes by using pioglitazone (Fig. 3m–o). Co-administered pioglitazone greatly intensified LAL expression in hepatocytes rather than Kupffer cells with an equal distribution over the periportal or perivenular regions. These results demonstrated that pioglitazone could enhance cytosolic lipolysis and autophagy mainly over hepatocytes. Hence, pioglitazone attenuated the hepatic steatosis may be mediated by enhanced lipophagy-related lipolysis, primarily over hepatocytes.

### Pioglitazone up-regulated gene expressions of cytosolic lipolysis, autophagy and β-oxidation differentially dependent on PPARα and PPARγ activation

**Figure 4:**
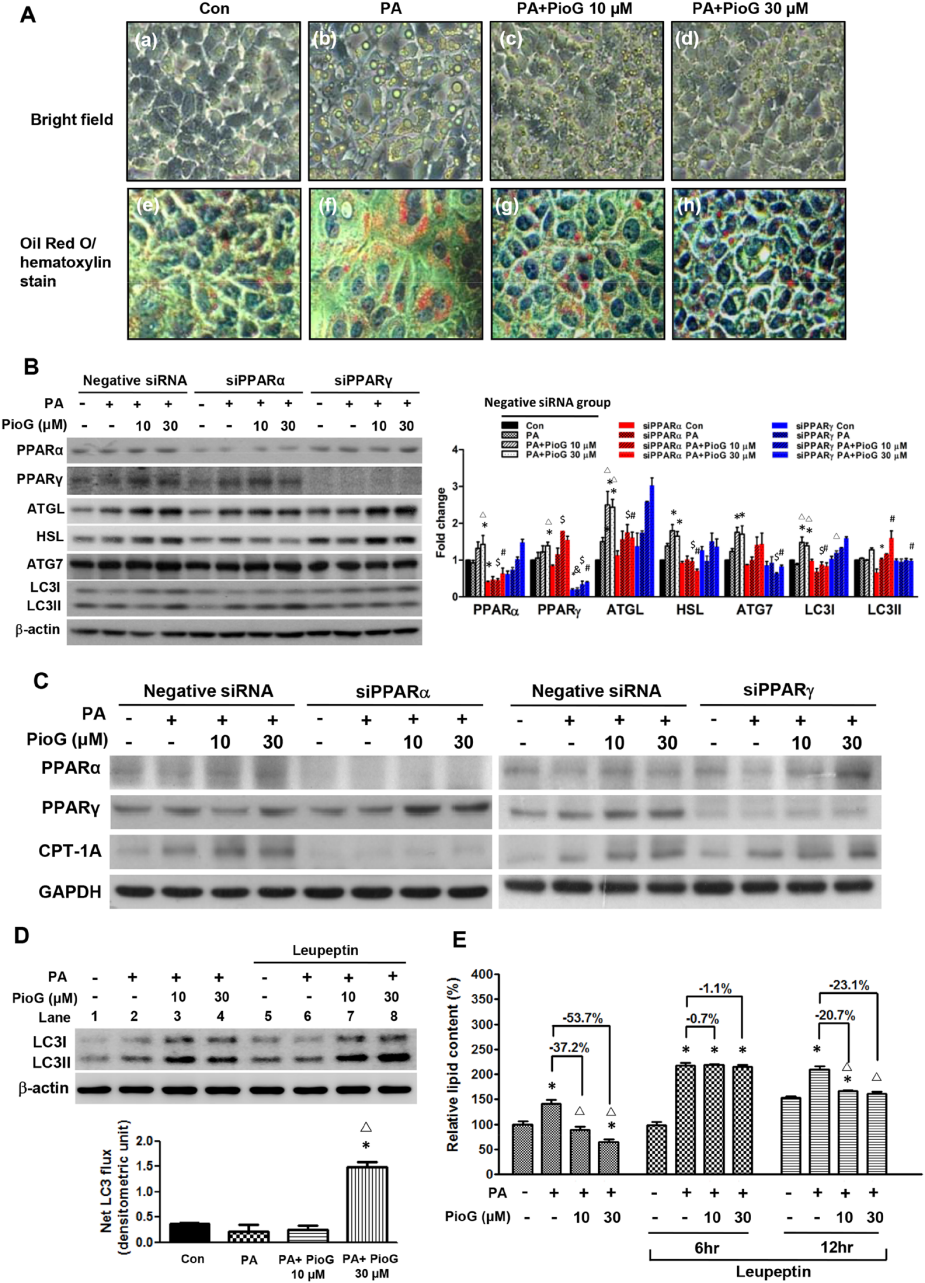
Pioglitazone ameliorated hepatic steatosis through enhancing lipolysis, β-oxidation, and autophagy dependent on PPARα and PPARγ activation. **(A)** AML12 cells treated with (a,e) control (b,f) 400 µM palmitic acid (PA), (c,g) 400 µM PA and 10 µM pioglitazone (PioG), (d,h) 400 µM PA and 30 µM pioglitazone. Cells with bright field and Oil-red O/hematoxylin stain to show lipid droplet. Magnification: 400X. **(B**,**C**) AML12 cells, transfected with siRNA against PPARα, PPARγ, and non-targeting negative siRNA, were treated with palmitic acid (PA, 400 μM) with or without pioglitazone (PioG) for 3 days. Protein expressions of the PPARα, PPARγ, ATGL, HSL, LC3, and CPT-1A were analyzed to control with GAPDH and β-actin. Data are expressed as fold change of the negative siRNA. *P* < 0.05 indicated as significant, *vs Control (con), ^Δ^vs PA, ^$^vs PA + PioG 10 μM, ^#^vs PA + PioG 30 μM. **(D)** Autophagic flux was determined by treating lysosomal inhibitor, leupeptin, for 4 hours to compare the LC3 expression with loading control of β-actin. Net LC3 flux was indicated by the difference of LC3II/β-actin ratio between leupeptin treated and untreated cells. Con: (lane 5-lane 1), PA: (lane 6-lane 2), PA + PioG10 μM: (lane 7-lane 3), PA + PioG 30 μM: (lane 8-lane 4). (**E**) AML12 cells, incubated with PA and pioglitazone as indicated for 48 hours, were then treated with leupeptin (100 µM) for 6 and 12 hours. The lipid content was determined by Oil-red O stain and quantified by reading absorbance at 492 nm. Data was expressed as fold or percentage change in mean + SEM. *P* < 0.05 indicated as significant, *vs Con, ^Δ^vs PA.

The alpha mouse liver 12 (AML12) cell treated with palmitic acid (PA) was established to elucidate the mechanism of cytosolic lipolysis, β-oxidation and autophagy regulated by pioglitazone. The AML12 cells treated with PA apparently resulted in LDs formation, whereas co-treatment with pioglitazone reduced the quantity and size of LDs by Oil Red O staining (Fig. 4A). This cell model was qualified as an *in vitro* hepatic steatosis model for further silent RNA assay to knockdown of PPARα and PPARγ.

Expressions of the ATGL, HSL, ATG7, and LC3 were significantly enhanced by pioglitazone (Fig. 4B). Enhanced expression of CPT-1A by pioglitazone is shown in Fig. 4C. The enhanced expressions of ATGL, HSL, and CPT-1A by pioglitazone were significantly impaired by knockdown of PPARα, but independent on PPARγ knockdown (Fig. 4B,C). Expression of the ATG7 was significantly decreased by PPARγ knockdown but independent on PPARα knockdown. Expression of the LC3II (active lipidated form) was still dose-dependently enhanced by pioglitazone by PPARα knockdown but kept steady by PPARγ knockdown (Fig. 4B). This result indicated regulation of the cytosolic lipolysis (ATGL, HSL) and β-oxidation (CPT-1A) were mainly dependent on PPARα activation, while the autophagy (ATG7 and LC3II) were primarily dependent on the PPARγ activation.

### Pioglitazone enhanced autophagy flux in mouse hepatocytes

LC3II was an active marker to indicate the component of autophagolysosomal membrane. To quantitatively determine the autophagic activity regulated by pioglitazone, net LC3 flux was measured by the difference from before and after treatment with the inhibitor of lysosomal protein degradation, leupeptin. The LC3II expression was significantly raised after inhibition by leupeptin. But the net LC3 flux is significantly increased more than 3 folds by adding 30 μM pioglitazone (Fig. 4D). The lipid accumulation in AML12 cells, initially treated by PA and pioglitazone for 48 hours, was dose-dependently attenuated by pioglitazone (Supplementary Fig. S1). The eluted lipid was then quantitatively determined by spectrophotometer (Fig. 4E). The leupeptin treatment for 6 hours completely blocked the lipophagy induced by pioglitazone. After two half-lives of leupeptin treatment (12 hours), pioglitazone significantly reduced the lipid accumulation again because of the diminishing inhibition of autophagy. Our result demonstrated that pioglitazone definitely enhanced the autophagic flux in hepatocytes.

**Figure 5:**
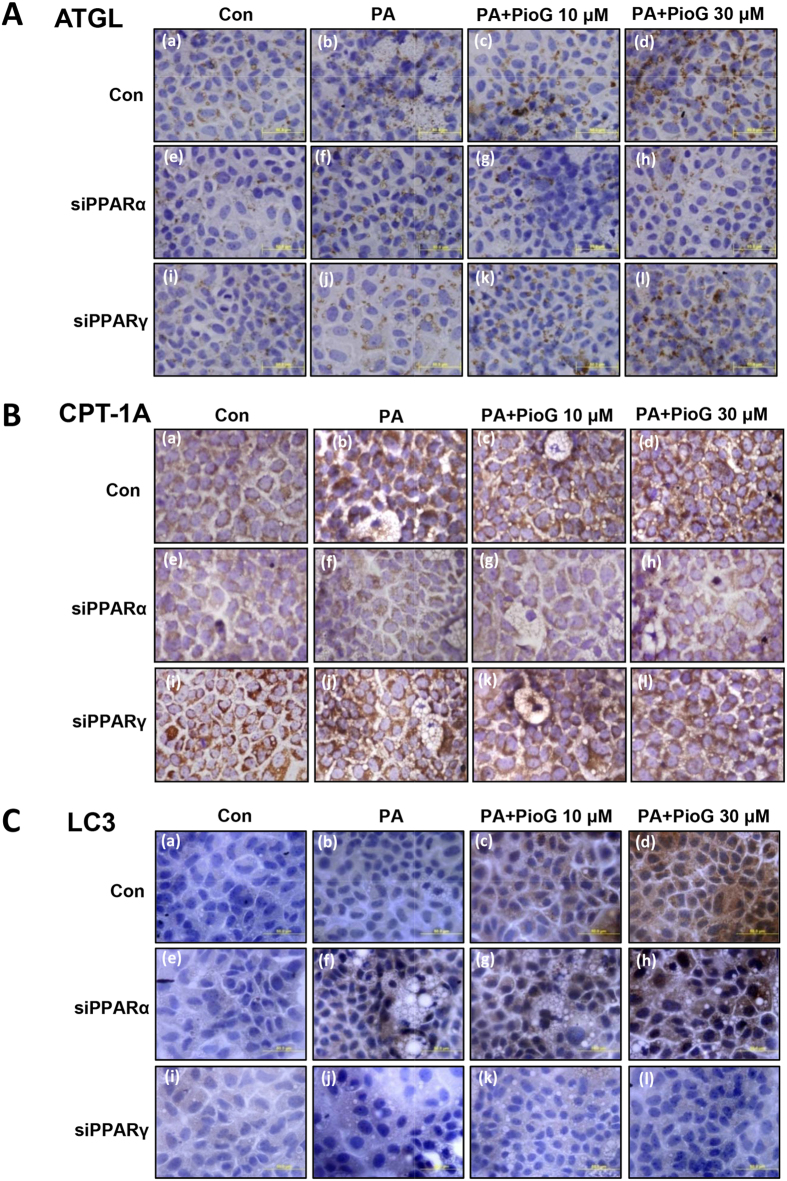
Immunochemical expressions of the ATGL, CPT-1Α and LC3 were differentially regulated by PPARα and PPARγ knockdown. AML12 cells were transfected with siRNA against PPARα, PPARγ, and non-targeting control siRNA, respectively. Then, the cells were treated with palmitic acid (PA, 400 μM) with or without pioglitazone (PioG, 10 or 30 μM) for 3 days. Protein expressions were analyzed by immunohistochemistry using antibody against (**A**) ATGL, (**B**) CPT-1Α, and (**C**) LC3, respectively.

To further confirm distribution of these proteins, ATGL, CPT-1 A and LC3 were determined by immunohistochemistry in cell block (Fig. 5A,B,C). The enhanced ATGL expression by pioglitazone was chiefly impaired by PPARα knockdown but not PPARγ knockdown (Fig. 5A). Expression of the CPT-1A was decreased significantly dependent on PPARα knockdown (Fig. 5B). Moreover, the increased expression of LC3 by pioglitazone was obviously diminished by PPARγ knockdown (Fig. 5C). These results, being consistent with the western blotting, revealed that pioglitazone augmented the processes of cytosolic lipolysis, β-oxidation and autophagy dependently on PPARα and PPARγ.

## Discussion

Pioglitazone can effectively improve steatosis, necroinflammation and slow the progression of fibrosis in NAFLD according to the outcomes of human clinical trial^23^. From the literatures, the plausible hepatoprotective effects of pioglitazone include enhancing insulin sensitivity, reducing lipid delivery to liver and muscle, increasing adiponectin production, and inhibition on both production of tumor necrosis factor-α by adipocytes and activation of macrophages^24^. However, the molecular mechanism of attenuating steatosis in liver was still unclear. Our result revealed that HFD could induce significant hepatic inflammation and steatosis. Co-administration of pioglitazone with HFD markedly lessened the hepatic triglyceride content, liver enzyme and serum insulin level. Pioglitazone enhanced cytosolic lipolysis, β-oxidation, lipophagy, and attenuated lipogenesis in liver, which occurred dominantly over hepatocytes. Our study evidenced an important clue that pioglitazone attenuated hepatic steatosis by amplifying cytosolic lipolysis, β-oxidation, and lipophagy, which were differentially mediated by both PPARα and PPARγ.

Development of the NAFLD is highly associated with insulin resistance and is also regarded as a hepatic manifestation of metabolic syndrome. It has been declared that peripheral insulin resistance triggers the lipolysis of adipose tissue, leading to increased free fatty acid influx to liver and then developing hepatic steatosis. Besides, hyperinsulinemia or insulin resistance has also been proved to impair autophagy and worsen the hepatic steatosis^25, 26^. Impaired hepatic autophagy is well known to be involved in obesity, diabetes and nonalcoholic steatohepatitis^21^. Yang *et al*. reported that down-regulation of the autophagy-related proteins (LC3, Beclin 1, Atg 5 and Atg 7) is significantly related to the development of obesity and insulin resistance in a HFD mouse model^27^. *In vitro* study has also shown that hepatic lipid content is increased in mice by *Atg 7* knockout, and the restoration of *Atg 7* expression markedly diminished endoplasmic reticulum (ER) stress and improved insulin sensitivity in mouse liver^17, 27^. These findings strongly support the hypothesis that an autophagy defect, especially a block in autophago-lysosomal fusion, does cause hepatic steatosis and insulin resistance^19, 28^. Inevitably, the functional alteration of autophagy results in the accumulation of the misfolded large molecules and dysfunctional cellular organelles, which may contribute to the development of endoplasmic reticulum (ER) stress and insulin resistance^29, 30^. The ER stress may aggravate the insulin resistance and the hepatic inflammation in a vicious cycle. Theoretically, interrupting the vicious cycle by decreasing the insulin level or activating lipophagy exhibits the benefits to alleviate the ER stress, improve insulin sensitivity and resolve metabolic syndrome-related abnormalities. This study clearly demonstrated pioglitazone steadily induced high expression of hepatic PPARγ, consistent with decreased serum insulin, activating autophagy and amplifying autophagy flux. Therefore, pioglitazone could diminish the hepatic steatosis that is proportionally mediated by improving insulin sensitivity and enhancing lipophagy. Subsequently, alleviation of hepatic steatosis may contribute to lessen hepatic inflammation, dampen hepatic ER stress and improve insulin sensitivity in an optimal cycle.

Expressions of ATGL and HSL, which normally present low level in mammalian liver, are abundant in adipose tissues to execute majority (~90%) of the cytosolic TG lipolysis. Over-expression of ATGL and HSL in cells other than adipocytes decreased LD size and TG content, whereas depletion of ATGL or HSL increased LD size and TG accumulation^31^. Both ATGL and HSL have proved to be responsible for ~43% of liver cytosolic TG hydrolase activity^32^. Using genetic and chemical ablation studies in a HFD mouse model, overexpression of hepatic ATGL and HSL promoted fatty acid oxidation to reduce hepatic TG contents by 40–60% and ameliorated hepatic steatosis. Previous evidence demonstrated hepatic ATGL mobilizes the fatty acid to β-oxidation through promoting the signaling of the PGC1α/PPARα activation^33, 34^. In a myoblast cell model treated with PPARα inhibitor, AMPK is considered as a downstream regulator of PPARα to activate ATGL^35^. Our study demonstrated a consistent result that pioglitazone significantly increased the expression of the ATGL and HSL, which are typically observed in hepatocytes. Regulation of the cytosolic lipolysis and β-oxidation by pioglitazone may depend on PPARα activation in mouse hepatocytes. However, this needs more study to assess the changes in metabolic assays.

Peroxisome proliferator-activated receptors (PPARs) are transcription factors acting as ligand-activators to regulate target gene transcription in lipid metabolism, energy homeostasis and inflammation^36^. PPARα is the major regulator to stimulate hepatic mitochondrial β-oxidation of fatty acid. It has been revealed that the high fat diet (fat composed of 50% energy, 12 weeks) significantly decreased hepatic PPARα expression (0.7 fold) with oppositely increased hepatic PPARγ expression (0.4 fold). Theoretically, activation of the PPARα, such as fenofibrate, could be a promising strategy to treat hepatic steatosis. Using fenofibrate has been reported to successfully ameliorate hepatic steatosis, improve insulin sensitivity in the mouse model of NAFLD, but failed on humans^37^. Hepatic PPARγ is normally expresses little as 9–12% of the expression in white adipose tissue. However, the patients with NAFLD exhibit abnormal high expression of PPARγ, which is related to up-regulation of lipogenesis and subsequent hepatic lipogenesis^38^. PPARγ activation by rosiglitazone is widely used to overcome insulin resistance to treat type 2 diabetic patients. As the speculated mechanism to stimulate hepatic lipogenesis, using rosiglitazone could cause damages on the liver. However, it has been evidenced that rosiglitazone exhibits the hepatoprotective effect in mouse HFD model and human studies, which is attributed by improving insulin sensitivity through cytokine effect (enhanced adiponectin and reduced tumor-necrosis factor-α). In clinical reality, using fenofibrate or rosiglitazone (PPARα/PPARγ activator) failed to achieve long-standing success in treating NAFLD or NASH^39^. The possible explanation for this could be side effects of increased oxidative stress secondary to β-oxidation by PPARα activation or up-regulation of lipogenesis by PPARγ activation. Pioglitazone, exhibiting dual PPARα/PPARγ agonist, has been established to improve insulin sensitivity and attenuate hepatic steatosis in human studies^22^. Our study presumed and evidenced a rational mechanism of pioglitazone to attenuate hepatic steatosis. In addition to improve the insulin sensitivity by pioglitazone, the molecular mechanism to benefit liver may be mediated by upregulation of the cytosolic lipolysis, β-oxidation and autophagy through PPARα/PPARγ activation. And agonist with dual PPARα/PPARγ activation may be superior to simply activate PPARα or PPARγ agonist to treat NAFLD.

PPARγ exists as two isoforms (γ1 and γ2) differing in their promoters and N-terminal sequences. PPARγ1 is ubiquitously expressed at a low level and PPARγ2 is intensely displayed in adipose tissue as a master regulator of adipogenesis. While expression at relatively low levels in liver, both PPAR isoforms are present in liver, particularly abundant in Kupffer cells^40, 41^. Activation of PPARγ1 was proved to have an anti-inflammatory function in macrophages. Specific PPARγ1 activated macrophages may attenuate tissue inflammation and enhance insulin signaling via an alternative pathway^42^. However, activation of PPARγ2 has been shown to enhance adipogenesis and lipogenesis-related gene expression and contribute to de novo lipogenesis and fatty liver formation in rodent models with HFD^24^. Nonetheless, our study evidently displayed pioglitazone augmented PPARγ1 activation may outweigh the effect of PPARγ2 activation. In this context, the hepatoprotective effect by pioglitazone may be supposed partly attributed to the anti-inflammatory effect of Kupffer cells via activation of PPARγ1.

Our results tried to explore the plausible mechanisms how pioglitazone biochemically and histologically improves the hepatic steatosis, which could provide molecular evidence to elucidate the hepatic benefit of pioglitazone on NAFLD treatment in human clinical trials. In conclusion, attenuation of the hepatic steatosis by pioglitazone may be conferred by refining insulin sensitivity, intensifying cytosolic lipolysis, β-oxidation and lipophagy-mediated lysosomal lipolysis in a manner of PPARα/γ dependently.

## Methods

### Animal experiment

Male C57BL/6 mice, obtained from BioLASCO Technology (Charles River Taiwan Ltd), were bred by standard procedures under the supervision of the Institutional Animal Care and Use Committee. The experiments were performed in the accordance with approved protocol by Institutional Animal Care and Use Committee of Kaohsiung Medical University. All of the procedures performed on mice were according to National Institute of Health guidelines. All mice (aged 7-weeks) were fed with standard chow diet for one week and then divided into three groups: (1) chow diet (n = 5) (basal diet TM 5755 with 10% fat, PMI nutrition International, ST. Louis, MO, USA); (2) HFD (30% of fat providing 53.1% of calories) (n = 5) (catalog #7166, PMI Nutrition International, St. Louis, MO, USA); (3) HFD combined with daily gastric gavage with pioglitazone 100 mg/kg/day (n = 8). Pioglitazone was kindly provided by Takeda Chemical Industries Ltd. (Taiwan). As the drug metabolized in mice are faster than human, our dosage was estimated equivalently to human dose according to the instruction leaflet from Takeda Company. The HFD, based on basal diet 5755, contained 40.6% carbohydrate (dextrin 23.6% and sucrose 15%), 15% corn oil, 15% lard, 19% protein, 4.3% fiber, 5% mineral mixture and 0.2% vitamin mixture. The experimental diets were continued for 8 weeks before sacrifice.

### Cell culture and treatment with pioglitazone

The AML12 (alpha mouse liver 12) liver cell line, obtained from Bioresource Collection and Research Center (BCRC, Taiwan), was cultured in 90% 1:1 mixture of Dulbecco’s modified Eagle’s medium and Ham’s F12 medium (Gibco, Grand Island, USA) with 0.005 mg/ml insulin, 0.005 mg/ml transferrin, 5 ng/ml selenium (ITS mixture, Gibco), 40 ng/ml dexamethasone (Sigma-Aldrich, St. Louis, MO) and supplemented with 10% fetal bovine serum (Gibco) at 37 °C in a humidified atmosphere with 5% CO_2_. For induction of hepatic steatosis *in vitro*, cells were treated with palmitic acid (Sigma-Aldrich, St. Louis, MO) 400 mM in DMSO and mix with 7.5% BSA in a ratio of 1:1 to incubate at 37 °C for 1 h. Cells were treated with 400 µM palmitic acid/BSA mixture for 3 days to induce hepatic steatosis. Pioglitazone (Sigma-Aldrich, St. Louis, MO) was dissolved in DMSO to make a 50 mg/ml stock for cell treatment. To assess autophagic flux, cells were treated with 100 µM leupeptin (Sigma-Aldrich, St. Louis, MO) for 4 hrs to inhibit the lysosomal proteins degradation.

### Small interfering RNA

The small interfering RNA (siRNA) of PPARγ, PPARα and non-targeting negative control were purchased from Dharmacon Inc. (Lafayette, CO, USA). Before transfection, the cells were plated in 6 well plates and incubated at 37 °C with 5% CO_2_. After incubating for 24 hrs, the cells displayed about 60% of confluence, and were then transfected using DharmaFECT® 1 transfection reagent (Dharmacon Inc.) according to the manufacture’s protocol.

### Biochemical analysis

Body weight was recorded weekly throughout the experiment. The animals were sacrificed after overnight fasting at the end of the experiment. A blood sample was drawn by heart puncture to measure serum glucose, triglyceride, and alanine aminotransferase with an autoanalyser (Roche Diagnostics, Taipei, Taiwan). Serum NEFA was determined by colorimetry with a commercial kit (Wako Pure Chemical Industries, Ltd, Osaka, Japan). Hepatic lipid content was extracted by the Folch method and determined with an autoanalyser (Roche Diagnostics, Taipei, Taiwan). Liver tissue was prepared in pieces and stored for protein extraction and immunohistochemical analysis.

### Protein extraction and Western blot

The cells were lysed with gently shaking in M-PER^®^ Mammalian Protein Extraction Reagent (Thermo Scientific^TM^, Rockford, IL, USA) or RIPA buffer for 5 min. The protein extracts were harvested by centrifugation at 13500 x g for 15 mins to pellet the cell debris. Thirty micrograms of protein was separated on a 10~15% SDS-polyacrylamide gel running with constant current for 2.5 hrs. After electrophoresis, the proteins were transferred to a PVDF membrane (Millipore, Bedford, M.A, USA), assembled in a Bio-Rad Transblot 100 V for 1 h. The membrane was then immersed in 5% skim milk at room temperature for 1 hour followed by incubated with primary antibody against mTOR (OriGene Technologies, Inc., Rockville, MD, USA), Beclin-1, PI3-Kinase Class 3 (Vps 34) (rabbit monoclonal antibody, Epitomic, Inc., Burlingame, CA, USA), Atg 7 (Epitomic, Inc., Burlingame, CA, USA), (OriGene Technologies, Inc., Rockville, MD, USA), LAL (SevenHills Bioreagents, Inc., Cincinnati, OH, USA), ATGL (Epitomics, Inc., Burlingame, CA, USA), HSL (Cell Signaling Technology, Inc., MA, USA), ACC1 (Millipore, Billerica, Massachusetts, USA), FAS (Abcam Inc., MA, USA), CPT-1A (Abcam, Cambridge, UK), PPARγ2/γ1(Chemicon International, Inc., USA), and PPARα (Millipore, Billerica, Massachusetts, USA). The expression of glyceraldehyde-3- phosphate dehydrogenase (GADPH, Cell Signaling Technology, Inc., MA, USA) was used as a control. The secondary antibody was HRP-conjugated rabbit-anti-mouse IgG (Abcam, Canbridge, MA, USA) and goat-anti-rabbit IgG antibodies (Jackson ImmunoResearch Laboratories, West Grove, PA). Membrane was developed by ECL plus detection reagents (Amersham International, NJ, USA) for band detection using X-ray film (Fujifilm, Tokyo, Japan). Quantitative comparison of the chemiluminescent images was achieved by Image J.

### Real-time quantitative PCR

Total RNA was extracted using TRIzol (Invitrogen), and applied to cDNA synthesis using TaqMan MicroRNA Reverse Transcription Kit (ABI, Foster City, CA, USA). Real-time quantitative PCR were performed using MiniOpticon^TM^ real-time PCR system (Bio Rad, Hercules, California, USA) with a QuantiTect SYBR Green kit (Bio Rad). The PCR primers were designed and synthesized by Invitrogen (Table 2). All the results were quantified based on *β-actin* as an internal standard. The optimized PCR program was 95 °C for 3 min, followed by 40 cycles of 95 °C for 15 s, 57 °C for 30 s and 72 °C for 1 min.

**Table 2:** Primer used for real-time PCR experiment.

Gene	RefSeq		5′-3′
*Atg7*	NM_028835	Forward	TCCGTTGAAGTCCTCTGCTT
		Reverse	TCCTACCACTTGGAGTCACC
*Atgl*	NM_025802.3	Forward	CCTTAGGAGGAATGCCCTGC
		Reverse	AACCCACTGGTAGACGGAAG
*β-actin*	NM_007393	Forward	GAAATCGTGCGTGACATC
		Reverse	GGAAGGAAGAACCCATACC
*Cpt-1a*	NM_013495.2	Forward	GACTCCGCTCGCTCATTCC
		Reverse	TCGGGAGTTTGTCTAGACGG
*Hsl*	NM_010719.5	Forward	TATTCCTGCTGTGAGGGCAC
		Reverse	TCCTAACCTACCAAACCCCC
*Lal*	NM_001111100.1	Forward	GACCACTCCCGATGCAACTC
		Reverse	GACCACTCCTTGTGAGCCAG
*Lc3*	NM_025735.2	Forward	CTTCGGCTTCTGAGTCAAGAGGAG
		Reverse	GTGGTCGGTCGGATGGTGTAG
*Pparα*	NM_001113418.1	Forward	TGGCTGCTATAATTTGCTGTGGAG
		Reverse	CGTTGGTAGGTCTACTGTGGAAG
*Pparγ*	NM_011146	Forward	GGAAGCCCTTTGGTGACTTTATGG
		Reverse	CTGTAGGTTCTGTTGGACGACG

### Histologic and immunohistochemical analysis

The dissected liver specimens were fresh-frozen and fixed in Tissue-TekR O.C.T compound (Sakura Finetechnical Co., Tokyo, Japan) for Oil-red O staining. The other dissected specimens were embedded in paraffin and prepared by hematoxylin and eosin (H&E) stain for histopathologic comparison. The tissue distribution of cytosolic lipolysis and lipophagy-related lipolysis was evaluated by immunohistochemical (IHC) staining. The liver specimens were processed and incubated overnight at 4 °C with rabbit anti-Atg7, LC3, ATGL, HSL, and LAL antibodies (diluted 1:1500 in PBS) separately. DAKO EnVision System Labelled Polymer-HRP (DAKO, Denmark) was applied using DAB as the chromogen followed by Mayer’s hematoxylin counterstaining and mounting. Negative controls were obtained by replacing the primary antibody with non-immune serum.

### Quantification of the lipid content treatment by leupeptin

Cells were fixed with 10% formalin for 10 min at room temperature, and washed twice with PBS. After 5 min of pre-incubation with 60% isopropanol, cells were stained by Oil-red O (Sigma-Aldrich, St. Louis, MO) for 1 hour, and then washed twice with PBS to remove excess dye. The Oil-red O was then extracted by isopropanol and determined by microplate spectrophotometer with absorbance at 492 nm (BioTek Epoch, Winooski, VT, USA). Wells absent of cells was served as the blank, and cells treated with vehicle served as the negative control. Lipid content was indicated in percentage (%) and estimated by (OD treated cells-OD blank)/(OD negative control-OD blank) × 100%.

### Statistical analysis

All statistical analyses were done by SPSS 18.0 for Windows (SPSS Inc., Chicago, IL, USA). Values are presented as means ± S.E. Statistical significance was determined as *p* < 0.05 using the Kruskal-Wallis test among three groups or Student’s *t* test between two groups.

## Electronic supplementary material

Supplementary Information
