# Supplementary material for: Pioglitazone Enhances Cytosolic Lipolysis, β-oxidation and Autophagy to Ameliorate Hepatic Steatosis
Source: Sci Rep. 2017 Aug 22;7:9030. doi: 10.1038/s41598-017-09702-3 (PMC5567271; doi:10.1038/s41598-017-09702-3)
Supplement: Supplementary file 1 — Supplementary Information [file 41598_2017_9702_MOESM1_ESM.pdf]

Pioglitazone Enhances Cytosolic Lipolysis, β-oxidation and Autophagy to Ameliorate Hepatic Steatosis

Pi-Jung Hsiao[1, 4], Hsin-Ying Clair Chiou[1], He-Jiun Jiang[1], Mei-Yueh Lee[1, 4], Tusty-Jiuan Hsieh[2, 4], Kung-Kai Kuo[3, 4]

[1]Division of Endocrinology and Metabolism, Department of Internal Medicine; Kaohsiung Medical University Hospital; [2]Graduate Institute of Medicine, Kaohsiung Medical University; [3]Hepatobiliary Division, Department of Surgery, Kaohsiung Medical University Hospital; [4]School of Medicine, College of Medicine, Kaohsiung Medical University

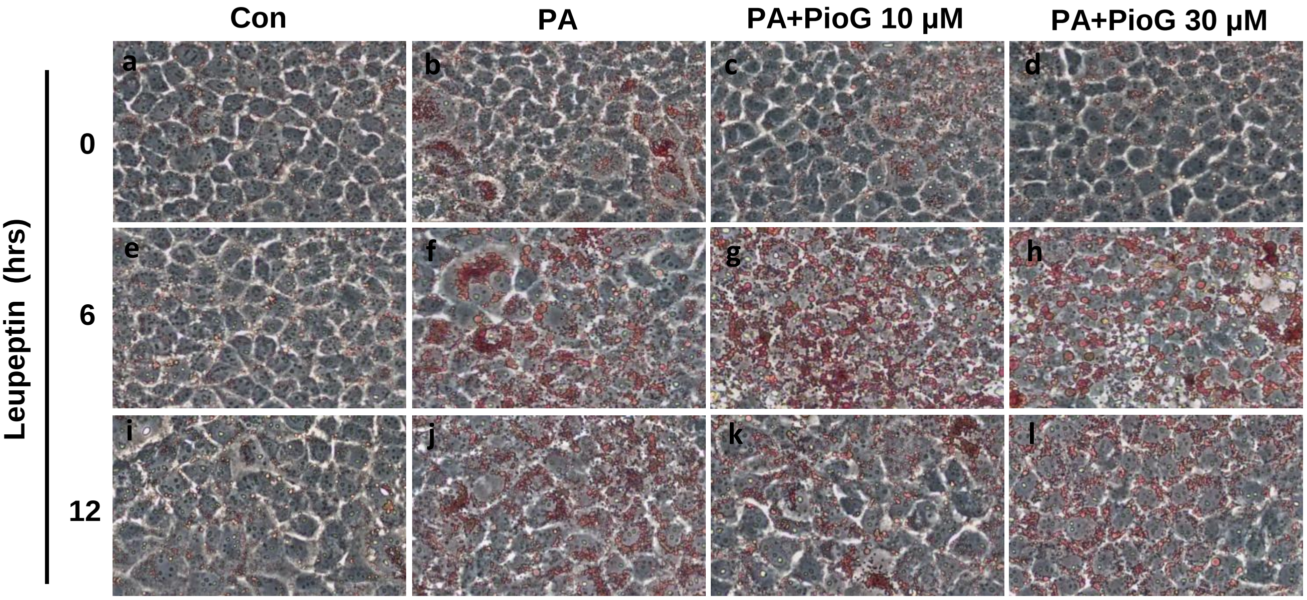

Supplementary Fig. S1: Leupeptin inhibits lipophagy induced by pioglitazone. AML12 cells, incubated with PA and pioglitazone as indicated for 48 hours, were then treated with leupeptin (100 μM) for 6 and 12 hours. The lipid content was determined by Oil-red O stain. Magnifications, 200X.
